# The role of nonverbal working memory in morphosyntactic processing by children with specific language impairment and autism spectrum disorders

**DOI:** 10.1186/s11689-017-9209-6

**Published:** 2017-07-04

**Authors:** Susan Ellis Weismer, Meghan M. Davidson, Ishanti Gangopadhyay, Heidi Sindberg, Hettie Roebuck, Margarita Kaushanskaya

**Affiliations:** 1 Department of Communication Sciences and Disorders, University of Wisconsin-Madison, Madison, WI 53706 USA; 2 Waisman Center, University of Wisconsin-Madison, Madison, WI 53706 USA; 3 Callier Center for Communication Disorders, University of Texas at Dallas, Dallas, TX 75235 USA

**Keywords:** Autism, Specific language impairment, Grammatical judgment, Working memory

## Abstract

**Background:**

Both children with autism spectrum disorders (ASD) and children with specific language impairment (SLI) have been shown to have difficulties with grammatical processing. A comparison of these two populations with neurodevelopmental disorders was undertaken to examine similarities and differences in the mechanisms that may underlie grammatical processing. Research has shown that working memory (WM) is recruited during grammatical processing. The goal of this study was to examine morphosyntactic processing on a grammatical judgment task in children who varied in clinical diagnosis and language abilities and to assess the extent to which performance is predicted by *nonverbal* working memory (WM). Two theoretical perspectives were evaluated relative to performance on the grammatical judgment task—the “working memory” account and the “wrap-up” account. These accounts make contrasting predictions about the detection of grammatical errors occurring early versus late in the sentence.

**Methods:**

Participants were 84 school-age children with SLI (*n* = 21), ASD (*n* = 27), and typical development (TD, *n* = 36). Performance was analyzed based on diagnostic group as well as language status (normal language, NL, *n* = 54, and language impairment, LI, *n* = 30). A grammatical judgment task was used in which the position of the error in the sentence (early versus late) was manipulated. A visual WM task (N-back) was administered and the ability of WM to predict morphosyntactic processing was assessed.

**Results:**

Groups differed significantly in their sensitivity to grammatical errors (TD > SLI and NL > LI) but did not differ in nonverbal WM. Overall, children in all groups were more sensitive and quicker at detecting errors occurring late in the sentence than early in the sentence. Nonverbal WM predicted morphosyntactic processing across groups, but the specific profile of association between WM and early versus late error detection was reversed for children with and without language impairment.

**Conclusions:**

Findings primarily support a “wrap up” account whereby the accumulating sentence context for errors positioned late in the sentence (rather than early) appeared to facilitate morphosyntactic processing. Although none of the groups displayed deficits in visual WM, individual differences in these nonverbal WM resources predicted proficiency in morphosyntactic processing.

**Electronic supplementary material:**

The online version of this article (doi:10.1186/s11689-017-9209-6) contains supplementary material, which is available to authorized users.

## Background

Grammatical deficits are a hallmark of children with specific language impairment (SLI) [1]. Additionally, a subset of children with autism spectrum disorders (ASD) have deficient grammatical abilities that have been described as being similar to those difficulties observed in SLI [2, 3]. There is evidence to suggest that both children with SLI [4–6] and children with ASD [7, 8] may have deficits in working memory skills in addition to their language challenges. Working memory (WM) refers to the processing of information while maintaining that information in temporary storage. Prior research has established the importance of WM in typical language processing by adults and children [9–13] as well as in children with language impairment [14–17]. While both verbal and nonverbal WM has been investigated in children with SLI and ASD, most of the research examining the association between WM and grammatical processing has employed verbal WM tasks. This makes it difficult to disentangle effects related to linguistic processing from those related to memory processes per se (see [18] for similar argument). Focusing on the possible link between visual WM and morphosyntactic processing serves to provide insight into the role of domain-general executive attention within WM with minimal influence from domain-specific verbal mechanisms [19]. The goal of this study was to investigate morphosyntactic processing on a grammatical judgment task in children with varying language abilities and to assess the association between grammatical judgment performance and nonverbal WM.

### Morphological deficits in children with SLI and ASD

Children with SLI have substantial difficulties with grammatical morphology; in fact, morphological deficits are typically considered to be a clinical marker of SLI [20–22]. Studies have shown that children with SLI have particular difficulty with marking finite verbs for tense and agreement [21, 23–28]. Deficits in noun morphology involving articles, plural markers and possessive markers have also been observed [29]. There is an extensive body of research documenting morphological deficits in children with SLI across a variety of languages (see [1] for review), and there are several theoretical accounts of SLI that attempt to explain the specific profile of grammatical challenges observed [21, 30, 31].

Although social communication (pragmatic) deficits rather than grammatical problems are the major hallmark of ASD, research has also noted morphosyntactic problems in children with ASD [32, 33]. Findings from several studies have suggested the morphological deficits in ASD resemble the types of grammatical deficits seen in SLI [2, 3, 34]. Whereas pragmatic deficits are inherent in ASD (as defined by DSM-5 [35]), it is generally agreed that only a portion of children with ASD have structural language deficits, affecting phonology, vocabulary, and/or grammar; therefore, various studies have subdivided children on the autism spectrum into those with language impairment versus those with normal structural language [2, 14]. An alternative view to the subgroup notion is that grammatical challenges are characteristic of ASD to varying degrees and weaknesses can be revealed on more complex tasks even in children who appear to have normal range abilities on standardized assessments [36].

### Working memory abilities in SLI and ASD

There is considerable evidence that children with SLI have deficits in verbal WM across a variety of tasks, including listening span measures [4–6, 15, 16, 37–39]. Findings regarding visuospatial WM in children with SLI are less consistent and likely reflect variations across studies in participants’ age, task demands, and comparison groups. Some studies have reported no significant limitations in nonverbal WM in SLI [5, 6], whereas other studies have found visuospatial WM deficits in children with SLI [40–44], including a meta-analysis of 21 studies that included complex (central executive) visuospatial WM tasks [45]. Recent research using latent variable analysis found that the underlying structure of WM was similar for children with and without SLI, with the different components of WM showing varying associations with language abilities [46]. The bulk of the findings indicate that when difficulties with visuospatial WM abilities are observed in SLI, the effects are less robust than in the case for verbal WM [45]. Children with SLI would be expected to perform worse on verbal WM measures given that the verbal domain is their primary area of deficit, but evidence that WM limitations extend to visuospatial tasks suggests domain-general impairments in SLI.

The evidence is decidedly mixed regarding verbal and visuospatial WM deficits in children with ASD. Contrary to the profiles typically seen in children with SLI, several studies have reported that individuals with ASD had visuospatial WM deficits but no verbal WM deficits [47–49]. Conversely, other studies have found deficits in verbal WM in children with ASD [7, 14] or deficits in both spatial and verbal WM [50, 51]. Williams and colleagues [51] found that the memory profile in children with ASD was characterized by deficits in complex visual and verbal memory as well as spatial WM. In fact, spatial WM performance discriminated most accurately between the children with ASD and control children. Recently, Hill and colleagues [14] administered several measures of verbal and nonverbal WM to three groups of children matched on age, including children with ASD who had co-occurring structural language impairment (ALI), children with ASD who were ‘language normal’ (ALN), and children with specific language impairment (SLI). The ALI children had more verbal WM impairments than the ALN children, but the ALI and SLI children only differed on verbal narrative WM with the ALI performing worse than the SLI group. Hill et al. [14] also compared these same children on nonverbal working memory tasks, and found no significant group differences. This finding is consistent with other research reporting that children with ASD displayed spatial WM equivalent to typically developing (TD) controls [52]. In addition to domain of WM, task complexity and sample characteristics likely contribute to discrepant findings in working memory abilities in ASD (see discussion by [7, 50, 51]).

### Relation between working memory and language

There are various models of WM that have been proposed to characterize the ability to temporarily store and simultaneously manipulate information [37, 53–59]. WM is traditionally indexed via complex memory span tasks that focus on either verbal or visuospatial information. For example, verbal WM is often measured by listening span measures in which the individual must comprehend and respond to sentences of increasing length while also recalling the last word in each sentence. The most established model, proposed by Baddeley and colleagues [37], conceptualizes WM as a multicomponent, limited capacity system with distinct but interactive storage and control mechanisms. The central executive (CE) component is a domain-general attention-allocation mechanism that controls and coordinates different activities within WM. Control functions comprising the CE include various cognitive processes such as selective attention, inhibition, allocating and shifting attentional resources, updating information, and coordination of multiple tasks [60]. Other memory models posit a general WM system with limited attentional resources; these models focus more on executive and attentional control components of WM. The Embedded-Processes model [61] contends that WM reflects activation of information from long-term memory that is the current focus of attention. According to Engle’s view, WM capacity is limited by the ability to control attention such that individuals with greater WM capacity display better controlled attention [62]. The Time-Based Resource-Sharing model proposed by Barrouillet and colleagues asserts that WM span is determined by domain-general attention that must be shared between storage and processing activities in a serial, time-based manner [54, 63].

In sum, there are certain commonalities across the various models of WM. It is agreed that WM entails a multicomponent mechanism that supports storage and maintenance of information along with concurrent processing. WM models posit limited capacity storage as well as a resource limited attentional mechanism (or CE) that directs control processes which are assumed to play a crucial role in WM performance. Findings from confirmatory factor analyses and structural equation models in adults indicate that WM capacity measures largely reflect a domain-general factor which is a strong predictor of fluid intelligence, whereas short-term memory tasks are much more domain specific and are predictive of verbal or visuospatial reasoning [64]. Further, there is evidence from studies with adults to suggest that visuospatial WM and verbal WM tasks differ in the extent to which they draw on executive attention. Verbal WM has been shown to rely on both domain-general attention and domain-specific verbal processes (rehearsal), whereas visuospatial WM primarily reflects domain-general attention [19]. This same pattern has been shown for children for verbal WM [65]. In the current study, we employed a visual WM task to explore the role of domain-general executive attention within WM in morphosyntactic processing, with minimal influence from domain-specific verbal processes.

Following prior research, described below, the current study examines the interplay between WM and language within the context of a grammatical judgment task. The ability to make grammatical judgments (which involve linguistic awareness) imposes particular WM demands with respect to executive control in order to allocate and shift attention to detect the location of morphological errors while processing the linguistic content and maintaining information in temporary storage. In typically developing children a significant relationship has been reported between verbal WM and grammatical judgment accuracy for various constructions (e.g., regular past tense, present progressive) [12]. Noonan et al. [66] demonstrated that WM capacity—as indexed by both verbal and visuospatial complex memory span measures—influenced language processing in children with language impairment independently from language competencies. That study used a composite measure of WM so it was not possible to determine the role of visuospatial WM independent of verbal WM. The present study will address that question.

Previous research has demonstrated a clear link between WM and language abilities in SLI [46, 67, 68], with stronger links observed for verbal than for nonverbal WM. Studies have demonstrated associations between verbal WM capacity and complex sentence comprehension [13, 68] and the effect of morphological/syntactic complexity on WM performance [69, 70]. Leonard and colleagues [71] examined memory abilities (verbal and spatial/short-term and WM) and processing speed relative to language abilities in a large sample of adolescents with and without SLI. Their findings from latent variable regressions indicated that speed of processing and memory abilities accounted for 62% of the variance in concurrent language scores, with verbal WM making the largest contribution. It is noteworthy that nonverbal (spatial) WM was not a significant predictor of language abilities in SLI in that study [71]. Conversely, Karasinski and Ellis Weismer [72] found that that spatial WM was a significant predictor of distant inference accuracy for adolescents with SLI in a spoken narrative comprehension task; although, verbal WM (listening span task) was a stronger predictor and remained significant after accounting for the effects of language and nonverbal cognition. Furthermore, moderator analyses reported by Vugs et al. [45] indicated that visuospatial WM was related to language impairment in children with SLI, with impairment in the storage component (rather than the central executive) driving the relationship.

Prior research has also examined the relationship of working memory to language abilities in children with ASD. Hill et al. [14] found that for a combined sample of ASD children with and without language impairment, the degree of language impairment was significantly related to verbal WM abilities, but was not significantly related to nonverbal WM abilities. A combination of spatial and verbal WM tasks was found to account for significant variance in language abilities (as well as autism severity) in children with high functioning autism (HFA) [50]. However, other studies have failed to find a significant relationship between measures of working memory and verbal abilities in children with ASD [8, 47, 73]. It is possible that these conflicting results may be attributable to task differences as well as level of language abilities of the participants in each sample [14, 74].

### Working memory and grammatical judgment tasks

Numerous studies have demonstrated that verbal WM resources are recruited during syntactic processing, especially when syntactic processing is tapped by grammatical judgment tasks [12, 75]. Grammatical judgment tasks require that individuals determine whether a sentence is grammatical or ungrammatical for specific grammatical structures of interest. There is substantial evidence that increased WM capacity is associated with better and faster detection of grammatical errors. This link has been established primarily for verbal WM [12, 15, 76] though there is some indication that visuospatial WM may also be implicated [66].

Several studies have employed grammatical judgment tasks in which the position of the error within the sentence has been manipulated in order to make inferences about the role of WM in syntactic processing. There are two competing theoretical perspectives motivating this research that lead to differing predictions. According to the “working memory” account [66, 77], errors that occur later in a sentence should be more difficult to detect than those occurring earlier because they place more demands on WM. That is, more information must be processed, integrated, and maintained as the sentence unfolds for late appearing errors (rather than early errors), making it more likely that WM capacity will be exceeded. Thus, the WM account predicts that early errors should be easier and faster to detect than late errors. On the other hand, the “wrap-up” account [78] emphasizes the facilitating effects of sentence context in syntactic processing, and would suggest that detection of errors occurring later in a sentence rather than earlier should benefit from “wrap-up effects”. The ability to utilize context by accumulating and integrating information as the sentence unfolds serves a buffering effect to reduce WM load; thus, contextual information provides less support for early errors than late errors and early error detection should depend on WM more than late error detection. Contrary to the WM account, the wrap-up account predicts better processing of late than early errors.

Noonan and colleagues [66] examined the influence of error position on judgments of grammaticality by school-aged children with SLI but no WM impairment and children with both SLI and WM impairment compared to two controls groups—one matched on age, nonverbal intelligence, and composite WM scores and one matched on age and nonverbal intelligence, respectively. WM status was determined based on a composite score from a standardized battery of well-validated memory measures that included verbal as well as visuospatial WM tasks. Results indicated that children with co-occurring language and WM impairments were significantly worse than controls at detecting late (but not early) errors. The interpretation of these findings was consistent with the WM account in that the investigators suggested that the SLI plus WM impairment group performed most poorly in this condition due to the fact that late errors entailed the greatest WM demands. However, it is noteworthy that the SLI group without WM impairment did not display an effect of error position and the TD controls were more accurate at detecting late than early errors on this grammatical judgment task.

In contrast, Blackwell and Bates [78] proposed the wrap-up account to explain their findings regarding adults’ performance on a grammatical judgment task. Using a dual-task paradigm, a secondary digit-span task was added to the primary task involving a grammatical judgment measure; under these conditions of increased cognitive load there was a disproportionate decrease in errors occurring early in the sentence rather than those occurring late in the sentence. Similarly, Wulfeck et al. [28] found that children with SLI, focal brain lesion, and TD controls were more accurate and faster at detecting errors late in the sentence rather than early in the sentence. These results were interpreted as indicating that, despite overall differences in grammatical sensitivity, all groups were capable of taking advantage of the accrual of linguistic information toward the end of the sentence as proposed by the wrap-up account.

### Current study

In summary, children with SLI as well as a subset of children with ASD are known to have deficits in morphosyntactic processing. There is mixed evidence regarding WM deficits in these groups. While there has been prior research examining the role of WM (particularly verbal WM) in grammatical processing by children with SLI, there is a dearth of information on this issue for children with ASD. Recently, there has been an increasing interest in exploring the extent of overlap in language (and cognitive) phenotypes for SLI and ASD [79–81]. To our knowledge, this is the first cross-group comparison to examine the role of WM resources in morphosyntactic processing by children with SLI and ASD.

Although we might expect that both of the groups with neurodevelopmental disorders would perform more poorly than controls on the grammatical judgment task, it is possible that the two groups may exhibit different patterns of response, implicating varying underlying cognitive processes (as has been claimed for comparisons of SLI and ASD groups on nonword repetition tasks tapping short-term memory [14, 82]). For instance, we might see differences between SLI and ASD groups with respect to detection of late vs. early errors given the purported difficulties of individuals with autism in integrating contextual information into a coherent whole [83, 84]. Problems with central coherence (using accumulating linguistic context) would be assumed to be a characteristic of the ASD phenotype (diagnosis linked) and would presumably impose an increased load on WM for these children. Whereas difficulties with various components of WM have been implicated in children with both SLI and ASD, limitations in central coherence would only be hypothesized for the ASD group. It is reasonable to assume that extant structural language abilities also play a role in syntactic processing as indexed by grammatical judgment tasks, and we know that some children with ASD have normal range structural language skills and others display language impairment [2]. Therefore, performance on the grammatical judgment task was examined both by diagnostic group (TD, SLI, ASD) as well as language status [TD + ASD “language normal” (ALN) vs. SLI + ASD language impairment (ALI)].

It can be argued that it is complicated to isolate the role of WM in grammatical processing in groups with language disorders when verbal measures of WM are used. Therefore, in the present study, we utilized a nonverbal WM measure to avoid confounding linguistic processing and WM. Further, we were interested in looking beyond connections between verbal WM and linguistic processing to determine the extent to which visual WM and executive attention processes may be implicated in morphosyntactic processing. We examined group differences in grammatical judgment and nonverbal WM, and we then assessed the extent to which nonverbal WM predicts morphosyntactic processing for different groupings of the sample based on diagnosis or language status. In a previous study, we addressed the issue of whether nonverbal WM contributes differentially to the detection of early vs. late errors in two groups of TD children (monolinguals vs. bilinguals) who both had intact WM skills but who differed in language-specific abilities [74]. Findings indicated that higher nonverbal WM was associated with greater sensitivity to morphosyntactic errors in bilinguals, but not monolinguals, suggesting that nonverbal WM skills may be more closely linked to syntactic processing in individuals with relatively lower linguistic abilities. Therefore, for the present study, we hypothesized that the relationship between nonverbal WM and morphosyntactic processing would be stronger for children with language impairment than for children with normal language.

In summary, our research questions for this study were:

1. Do groups of children defined on the basis of diagnostic category (ASD, SLI, TD) or language status (ALI + SLI or ALN + TD) differ in the accuracy and/or speed with which they detect early vs. late errors in a grammatical judgment task?
2. Do these groups differ in nonverbal WM skills?
3. What is the association between morphosyntactic processing and nonverbal WM within groups identified by diagnostic category or language status?

## Methods

### Participants

The participants in the present study were enrolled in a larger project investigating the association between executive function (EF) and language in school-age children with differing language backgrounds: TD monolingual English speakers, TD bilingual English-Spanish speakers, children with ASD, and children with SLI. Only the TD, ASD, and SLI groups are the focus of the current report. These subgroups also completed a lexical processing task and various EF measures as previously reported [85]. The TD children in this study comprise a subset of the sample in a prior investigation of morphosyntactic processing by monolingual versus bilingual children [74].

Children in all groups were recruited through local schools, community centers, and clinics using flyers and website postings. In addition to these approaches, the children with ASD were recruited through a research registry at the Waisman Center consisting of families who had indicated an interest in having their child participate in research studies. The ASD and SLI groups, as well as the TD group, represented convenience samples based on adult referrals and willingness of the children to participate. Parents of children in each of the groups provided written consent for their child to participate, and all study procedures were approved by the university’s Institutional Review Board. All of the children in this study were monolingual English speakers. Based on parent report, 72**%** of the children in the study were Caucasian, 14**%** were African American, 11% were multiracial, 2**%** were Asian, and 1**%** were American Indian. In terms of ethnicity, 10**%** were Hispanic/Latino and 90**%** were not Hispanic/Latino. Children had normal (or corrected to normal vision) by parent report and all passed a hearing screening at 20 dB at 1000, 2000, and 4000 Hz at the time of the assessment.

A total of 84 children were enrolled in the current study: 36 TD (18 males), 27 ASD (23 males), and 21 SLI (12 males). The three groups were drawn from a larger participant pool such that the diagnostic groups were comparable in terms of chronological age (*p* = .32**)**, nonverbal IQ (*p* = .12), and SES (*p* = .99); see Table 1. Nonverbal IQ was assessed using the Perceptual Reasoning Index of the *Wechsler Intelligence Scale for Children*, 4th Edition (WISC-IV) [86], which is comprised of the Block Design, Picture Concepts, and Matrix Reasoning subtests. Number of years of maternal education was used as the index of SES.

**Table 1:** Participant characteristics (means and standard deviations) for the diagnostic groups (TD, ASD, SLI) and language status groups (NL, LI)

	TD Group (*n* = 36)	ASD Group (*n* = 27)	SLI Group (*n* = 21)	NL Group (*n* = 54)	LI Group (*n* = 30)
Age (years)	9.5 (1.0)	9.6 (1.2)	9.9 (1.1)	9.5 (1.1)	9.8 (1.0)
Nonverbal IQ^a^	106.8 (10.3)	107.6 (13.9)	101.4 (7.4)	108.3 (12.0)	100.3 (7.8)
SES^b^	16.2 (2.8)	16.2 (3.2)	16.2 (4.6)	16.4 (3.0)	15.9 (4.0)
Language Comprehension^c^	108.4 (13.5)	90.1 (19.9)	81.5 (9.9)	105.8 (13.8)	77.5 (12.1)
Language Production^d^	107.9 (12.8)	90.9 (20.7)	77.7 (9.4)	106.4 (12.5)	74.7 (11.2)
Core Language^e^	107.2 (12.3)	89.1 (21.0)	76.8 (7.2)	105.4 (11.9)	73.2 (10.8)
Social Communication^f^	4.1 (4.1)	18.7 (6.7)	7.7 (5.4)	8.9 (8.5)	11.4 (7.8)
Race/Ethnicity	24 White	24 White	12 White	41 White	19 White
	6 Black	0 Black	6 Black	6 Black	6 Black
	1 Asian	0 Asian	1 Asian	1 Asian	1 Asian
	0 Nat Am^g^	0 Nat Am	1 Nat Am	0 Nat Am	1 Nat Am
	5 Multi^h^	3 Multi	1 Multi	6 Multi	3 Multi
	1 Hisp/Latn^i^	4 Hisp/Latn	3 Hisp/Latn	2 Hisp/Latn	6 Hisp/Latn

Note: *TD* typical development, *ASD* autism spectrum disorders, *SLI* specific language impairment

^a^Wechsler Intelligence Scale for Children-4th Edition Perceptual Reasoning Composite Score

^b^Socioeconomic status based on mother’s years of education

^c^Clinical Evaluation of Language Fundamentals-4th Edition (CELF-4) Receptive Language Index Score

^d^CELF-4 Expressive Language Index Score

^e^CELF-4 Core Language Score

^f^Social Communication Questionnaire score

^g^Nat Am = Native American

^h^Multi = multiracial/other

^i^Hisp/Latn = Hispanic/Latino

All of the children in the ASD group had previously received a clinical or educational diagnosis within their home communities (by a pediatrician, developmental psychologist, school psychologist or an interdisciplinary team). At the time of assessment for this study, the community diagnosis was confirmed by an experienced clinical psychologist using the *Childhood Autism Rating Scale*, Second Edition for high functioning individuals (CARS2-HF) [87]. A minimum cutoff score of 25 was used as this corresponds to the 10th percentile of CARS2-HF scores among individuals with ASD in the standardization sample. CARS2-HF scores for the ASD group were as follows: *M* = 44.8, *SD* = 4.6, range 37 to 53. All groups were administered the *Social Communication Questionnaire* (SCQ) [88], a parent-report autism screening measure. The TD and SLI groups scored significantly lower than the ASD group, indicating that they did not display behaviors suggesting concerns regarding autism spectrum disorders (see Table 1). Language abilities for the ASD group varied considerably as summarized in Table 1. There were 9 (out of 27) children within the ASD group who met the SLI criteria of at least 1.25 SDs below the mean on a standardized language measure (described below) and 11 (out of 27) who scored at least 1 SD below the mean.

Children in the SLI group were assessed with a standardized language measure to confirm an SLI diagnosis; they scored at least 1.25 SD below the mean on one or more summary scores from the *Clinical Evaluation of Language Fundamentals, Fourth Edition* (CELF-4) [89]: core language, receptive language, or expressive language. In addition to meeting this criterion, children in the SLI group obtained a core language score (combination of receptive and expressive language scales) of at least 1 SD below the mean, indicating that they had some extent of language delay in both language comprehension and production. It was not possible to obtain a core language score for one child with SLI because she did not complete the expressive portion of the CELF-4 but her language impairment was confirmed by a standard score of 79 on the receptive portion of the test. Nine of the 22 children (41%) in the SLI group were currently receiving language intervention through their schools. In contrast to the children with SLI, children in the TD group had no history of language delay or intervention and all performed within 1 SD of the mean or higher on core language scores on the CELF-4.

The same sample of 84 children was also grouped on the basis of language status. By splitting the groups based on language abilities across diagnostic categories, it is possible to make a stronger case that performance is related to linguistic skill rather than anything integral to diagnosis of TD, SLI, or ASD. That is, this grouping ostensibly provides a “diagnosis neutral” look at children’s performance by only considering their language capabilities. Thus, two groups were formed to compare those with normal language (NL) to those with language impairment (LI). The NL group (*n* = 54; 34 males) consisted of children diagnosed as TD as well as children with ASD who had age appropriate structural language abilities (i.e., TD + ALN), whereas the LI group (*n* = 30; 19 males) consisted of children with SLI and children with ASD who had language impairments based on our criteria (i.e., SLI + ALI). The Language Status groups did not differ significantly in terms of age (*p* = .23) or SES (*p* = .47), but did differ on nonverbal IQ, *F*(1,82) = 9.35, *p* = .003 (even though both groups demonstrated normal range abilities); see Table 1.

### Procedure

Children were assessed by experienced examiners, including a certified speech-language pathologist and clinical psychologist (for the SLI and ASD groups). Sessions were conducted in child-friendly testing suites in a research laboratory. Evaluations were completed in two sessions lasting approximately 2 h each.

### Tasks

#### Nonverbal working memory

**Fig. 1:**
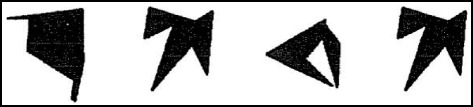
Sample abstract figures used as the visual stimuli for the N-back task

A visual N-back task was used as the measure of nonverbal WM. This computerized task was presented using E-Prime Studio 2 [90]. Children were instructed to match the shape that appeared on the computer screen to shapes that they had viewed previously [91, 92]. Stimuli for the N-back task were comprised of abstract shapes that are difficult to label [93, 94], reducing the likelihood that children would use verbal mediation or rehearsal strategies (see Fig. 1). There were three conditions of increasing difficulty in the N-back task: 0-back, 1-back, and 2-back. For the 0-back condition, children were instructed to press the green button on a response box when they saw the target shape and to press the red button when a different shape appeared. In the other conditions, children were required to press the green button when the shape was the same as the one that appeared one (1-back) or two (2-back) trials before the target shape and to press the red button if the shape was different. Children completed five practice trials each for the 0-back and 1-back conditions and eight practice trials for the 2-back condition before completing the task. Across the three conditions, each stimulus was presented for 1500 ms, with an inter-stimulus interval (ISI) of 500 ms. The N-back task consisted of a total of 40 trials, including 10 “hits” (target shape/green button items) and 30 “misses” (non-target shape/red button items). A fixed pseudo-random presentation sequence was used such that there were at least two intervening trials between target shapes in the 0-back condition and the N-1 and N-2 sequences were not repeated more than 10 times. Overall accuracy across the three conditions was used as the index of WM for the N-back task.

#### Grammatical judgment

An adapted version of a grammatical judgment task developed by Wulfeck et al. [28] was used to assess morphosyntactic processing. This task involved having children listen to sentences in order to determine whether they were grammatically correct or incorrect. Our version of the task focused on errors of omission because children with language impairment make more zero marking errors (drop grammatical morphemes) than errors of other types [1, 3, 95]. We targeted omission of regular past tense –*ed* markers and auxiliary markers given the evidence that these are among the forms that are particularly problematic for English-speaking children with language impairment [96]. We manipulated the position of errors such that they occurred either early or late in the sentence (see Additional file 1 for sample stimuli; refer to Gangopadhyay et al., 2016 for full stimulus list). The stimuli for this study consisted of 56 sentences, 28 ungrammatical sentences, and 28 grammatical control sentences matched on length and equated as a set for syntactic complexity. All target verbs in ungrammatical and grammatical control sentences were ones that are typically mastered by 8 years of age based on grade-level norms. Within the grammatical and ungrammatical sentences there were four conditions—early control, early omission, late control, and late omission—comprised of seven sentences each. Prior to testing, six practice sentences with verbal feedback were administered to ensure that children understood the task.

The grammatical judgment task was designed using E-Prime Studio 2. Stimulus sentences were recorded in a sound booth and standardized to the same amplitude range. A buffer of 3 ms was added to the beginning and end of each sentence. Early errors occurred within the first 1200 ms of the sentence and late errors occurred after 1200 ms, per guidelines established by Wulfeck et al. [28]. The average point in the sentence where errors occurred was 0.96 s (SD = 0.12 s) for early errors and 2.61 s (SD = 0.54 s) for late errors. Thus, there was a 1.65 s difference between early and late errors, which is a significant difference *t*(13) = −11.92, *p* < .001*.* Auditory stimuli were presented in the sound field (via computer speakers), and participant responses consisted of button presses using a serial response box attached to a desktop computer. Children were instructed to press the button with the smiling face if the sentence “sounded good, like something a person would really say” or to press the button with the frowning face if the sentence “sounded bad, like something a person would not really say.” Children were instructed to press the button as soon as they decided if the sentence was grammatical or ungrammatical, but the sentence played to the end even if the response occurred earlier. The ISI between each sentence was set to 750 ms unless a response had not yet occurred and then participants were allowed up to 2000 ms to respond following the end of a sentence. A pseudorandomized order of presentation was employed such that no more than three grammatical or ungrammatical sentences occurred in sequence and sentences with the same ungrammatical structure did not occur together.

Accuracy scores were computed for each child for the grammatical and ungrammatical conditions. Sensitivity (*A’*) and response bias (*B”*) scores were calculated using Stanislaw and Todorov’s [97] procedure (see [74] for more details). A sensitivity score of 0.5 indicates an inability to distinguish between grammatical and ungrammatical sentences, whereas a score of 1 indicates perfect performance. Response bias scores provide an index of the child’s tendency to respond “yes” or “no,” such that a score of -1 indicates an extreme bias to say “yes” for each response and 1 indicates an extreme bias to respond “no.” Sensitivity and response bias scores have been used to obtain a more nuanced assessment of performance in prior research using Yes/No tasks [e.g., 86].

Reaction times (RTs) were recorded for both grammatical and ungrammatical sentences. To calculate RTs for ungrammatical sentences, it was necessary to take into account the point at which the grammatical violation occurred. Following Wulfeck et al. [28], the point of violation was defined as the offset of the word preceding the error. For ungrammatical sentences, the time to the point of violation was subtracted from the total RT in order to obtain true RTs. RT data were analyzed only for correct responses. RT data were trimmed to remove outliers at the level of each child so that trials were excluded that fell more than 2.5 SDs from that child’s mean RT for all ungrammatical sentences. RTs that were ≤0 ms were also eliminated because they occurred prior to the error in sentence. These criteria resulted in the elimination of a total of 1.9% of all trials.

### Analyses

For the grammatical judgment task, separate mixed-model ANOVAs were conducted on sensitivity (*A’*), bias (*B”*), and RT scores separately for the diagnostic group and language status comparisons. These involved a 2 × 3 ANOVA with error position (early vs. late) as the within-subjects variable and diagnostic group (TD/ASD/SLI) as the between-subjects variable or a 2 × 2 ANOVA (position × NL/LI) for the language status group. Given that the language status groups differed significantly with respect to nonverbal cognition, analyses of covariance (ANCOVAs) were also completed using WISC-IV composite scores as the covariate. We acknowledge the debate within the literature regarding the use of IQ scores as covariates in studies of neurodevelopmental disorders [98]; however, we presented both analyses (with and without the NVIQ covariate) to allow comparison with prior studies that used an IQ covariate. ANOVAs were used to assess group differences on the two nonverbal WM measures. Finally, simple linear regression analyses were performed separately for the diagnostic groups and language status groups to determine whether WM predicted morphosyntactic processing (*A’* and RT scores). We used Holm-Bonferroni sequential correction to account for Type I error in the multiple regression analyses conducted in this study. The adjusted *p* values were calculated using an online EXCEL calculator developed by Justin Gaetano [99].

## Results

### Grammatical judgment

The ANOVA for the diagnostic groups examining *A’* scores revealed a main effect of position, *F*(1,81) = 7.10, *p* = .01, *ŋ*_*p*_^*2*^ = 0.08. Children were better at detecting late errors (*M* = 0.78, *SE* = 0.02) than early errors (*M* = 0.73, *SE* = 0.02). There was also a main effect of group, *F*(2,81) = 5.63, *p* < .001, *ŋ*_*p*_^*2*^ = 0.12. Pairwise comparisons revealed that the TD group (*M* = 0.82, *SE* = 0.02) displayed significantly (*p* < .05) better sensitivity to grammatical errors than the SLI group (*M* = 0.69, *SE* = 0.03). The *A’* scores for the ASD group (*M* = 0.76, *SE* = 0.03) did not differ significantly from the scores of the other two groups. The position × group interaction was not statistically significant, *F*(2,81) = 2.38, *p* = .10, *ŋ*_*p*_^*2*^ = 0.06. Analysis of *B''* scores indicated that there was no significant difference for the diagnostic groups with respect to response bias, *F*(2, 81) = 0.54, *p* = .59, *ŋ*_*p*_^*2*^ = 0.01.

Analysis of RT scores for the diagnostic groups revealed a main effect of position, *F*(1,80) = 557.57, *p* < .001, *ŋ*_*p*_^*2*^ = 0.88, such that RTs for detecting early errors were longer (*M* = 2936.74, *SE* = 54.10) than RTs for detecting late errors (*M* = 1825.79, *SE* = 35.10). The main effect of group was not significant for RT, *F*(2, 80) = 1.23, *p* = .30, *ŋ*_*p*_^*2*^ = 0.03, nor was the position × group interaction effect, *F*(2,80) = 2.12, *p* = .13, *ŋ*_*p*_^*2*^ = 0.05.

For the language status groups, the analysis of *A’* scores yielded a significant main effect of position, *F*(1,82) = 8.70, *p* < .001, *ŋ*_*p*_^*2*^ = 0.10. Late errors were detected more easily (*M* = 0.77, *SE* = .0.01) than early errors (*M* = 0.72, *SE* = 0.02). There was also a significant main effect of group, *F*(1,82) = 22.65, *p* < .001, *ŋ*_*p*_^*2*^ = 0.22, with the NL group performing better (*M* = 0.82, *SE* = 0.02) than the LI group (*M* = 0.67, *SE* = 0.02). The position × group interaction was significant, *F*(1,82) = 6.97, *p* = .01, *ŋ*_*p*_^*2*^ = 0.08, indicating that the LI group performed better on the late errors (*M* = 0.72, *SE* = 0.02) than on early errors (*M* = 0.63, *SE* = 0.03) whereas the NL group performed similarly on late errors (*M* = 0.82, *SE* = 0.02) and early errors (*M* = 0.82, *SE* = .02). Analysis of *B'*' scores revealed that the LI and NL groups did not differ significantly with respect to response bias, *F*(1,82) = 0.36, *p* = .55, *ŋ*_*p*_^*2*^ = 0.00.

Because the language status groups differed significantly on nonverbal cognition, an analysis of covariance (ANCOVA) was conducted using WISC-IV scores as the covariate in addition to the original analysis of *A’* scores reported above. ANCOVA results revealed no significant main effect of position, *F*(1,81) = 0.58, *p* = .45, *ŋ*_*p*_^*2*^ = 0.01. However, there was a significant main effect of group, *F*(1,81) = 14.50, *p* < .001, *ŋ*_*p*_^*2*^ = 0.15 such that the NL group performed better (*M* = 0.81, *SE* = 0.02) than the LI group (*M* = 0.69, *SE* = 0.02). The position × group interaction was also significant, *F*(1,81) = 5.50, *p* = .02, *ŋ*_*p*_^*2*^ = 0.06. The LI group was better at detecting late errors *M = *0.73, *SE = *0.02) than early errors (*M* = 0.65, *SE* = 0.03) but the NL group performed equally well on late errors (*M *= 0.81, *SE* *​ = 0.02) * and early errors (*M* = 0.81, *SE* = 0.02). Analysis of *B''* scores from the ANCOVA again revealed no significant difference in response bias for the LI and NL groups, *F*(1,82) = 0.55, *p* = .55, *ŋ*_*p*_^*2*^ = 0.00.

In terms of RTs for the language status groups, ANOVA results revealed a significant effect of position, *F*(1,81) = 524.28, *p* < .001, *ŋ*_*p*_^*2*^ = 0.87, with longer RTs observed for early errors (*M* = 2944.84, *SE* = 56.13) than late errors (*M* = 1825.24, *SE* = 35.62). The main effect for language status group with respect to RTs was not significant, *F*(1,81) = 0.08, *p* = .77, *ŋ*_*p*_^*2*^ = 0.00 and the position × group interaction was also not significant, *F*(1,81) = 0.01, *p* = .94, *ŋ*_*p*_^*2*^ = 0.00**.** Statistically equivalent results for RTs were obtained from the ANCOVA analysis that accounted for group differences in nonverbal cognition. There was a significant position effect, *F*(1,80) = 9.73, *p* < .001, *ŋ*_*p*_^*2*^ = 0.11, such that children were slower to respond to early errors (*M* = 2942.23, *SE* = 56.72) than late errors (*M* = 1826.26, *SE* = 36.02). There was no main effect for language status group for RTs, *F*(1,80) = 0.04, *p* = .84, *ŋ*_*p*_^*2*^ = 0.00, or position × group interaction, *F*(1,80) = 0.03, *p* = .84, *ŋ*_*p*_^*2*^ = 0.00.

### Nonverbal WM

The ANOVAs examining performance of the diagnostic groups indicated that there was no significant group difference on the N-back task, *F*(2,81) = 0.57, *p* = .56, *ŋ*_*p*_^*2*^ = 0.01. On the N-back task the following values were obtained: TD *M* = 0.78, *SE* = 0.04, ASD *M* = 0.75, *SE* = 0.04, and SLI *M* = 0.82, *SE* = 0.05.

Similarly, analysis of the language status groups yielded no significant differences on the N-back task, *F*(1,82) = 0.21, *p* = .65, *ŋ*_*p*_^*2*^ = 0.00. Group means were NL *M* = 0.79, *SE* = 0.03, and LI *M* = 0.77**,** *SE* = 0.04.

### Relation between nonverbal WM and grammatical judgment

In order to assess the association between nonverbal WM and morphosyntactic processing, we employed separate linear regression models for each group within the diagnostic and language status groupings. The predictor variable was 1-back target accuracy on the N-back task (this specific index from the N-Back task was used in a latent variable analysis of EF [100]). The criterion variables were early *A’*, late *A’*, early RT, and late RT. Holm-Bonferroni sequential correction was used to account for the multiple regression analyses and adjusted *p* values (*p’*) are reported.

For the diagnostic groups, the N-back task did not significantly predict *A’* scores for early errors on the grammatical judgment task for the TD group, *F*(1,34) = 0.74, *p’* = .79, *R* ^*2*^ = 0.02 (see summary of regression findings in Table 2). For late errors, however, there was a significant relationship between WM and morphosyntactic processing for the TD group, *F*(1,34) = 8.58, *p’* = .02, *R* ^*2*^ = 0.20, such that N-back scores predicted performance. For the ASD group, nonverbal WM was associated with *A*’ scores for early errors on the grammatical judgment task, *F*(1,25) = 10.62, *p’* = .02, *R* ^*2*^ = 0.30, such that the N-back task significantly predicted performance. The same pattern was observed for *A'* scores for late errors in the ASD group, *F*(1,25) =11.10, *p′ =* .02, *R* ^*2*^ = 0.31. For the SLI group, the association between visual WM and *A’* scores for early errors was not significant, *F*(1,19) = 3.09, *p’* = ..29, *R* ^*2*^ = 0.25, and similarly there was no significant relationship for late errors, *F*(1,19) = 0.12, *p’* = .79, *R* ^*2*^ = 0.01.

**Table 2:** Summary of nonverbal working memory and morphosyntactic processing relationships for the diagnostic groups and language status groups

Diagnostic groups	*A’* early errors	*A*’ late errors	RT Early Errors	RT late errors
TD	NS	*R* ^2^ = 0.20*	NS	*R* ^2^ = 0.22*
ASD	*R* ^2^ = 0.30*	*R* ^2^ = 0.31*	NS	NS
SLI	NS	NS	NS	NS
Language status Groups				
NL	NS	*R* ^2^ = 0.20*	NS	NS
LI	*R* ^2^ = 0.21*	NS	NS	NS

Note: *A*’ sensitivity, *RT* reaction time, *TD* typical development, *ASD* autism spectrum disorders, *SLI* specific language impairment, *NL* normal language, *LI* language impairment, *NS* not significant

*p ^′^ < .05 after Holm-Bonferroni correction

Next, we examined the role of nonverbal WM in predicting RT scores for early vs. late errors for the diagnostic groups. For the TD group, there was not a significant relationship between WM and RT scores for early errors, *F(*1,34) = 2.95, *p’* = .38, *R* ^*2*^ = 0.08. However, the association between nonverbal WM and RTs for late errors for the TD group was significant, *F*(1,34) =9.75, *p’* = .02, *R* ^*2*^ = 0.22. For the ASD group, nonverbal WM did not predict RT on errors occurring early in the sentence, *F*(1,25) =5.31, *p’* = .15, *R* ^*2*^ = 0.18 or late in the sentence, *F*(1,25) = 0.08, *p’* = .99, *R* ^*2*^ = 0.00. Similarly, for the SLI group there was no significant relationship between WM and RTs for either early errors, *F*(1,18) = 0.02, *p’* = .99, *R* ^*2*^ = 0.00, or late errors, *F*(1,19) =0.01, *p’* = .99, *R* ^*2*^ = 0.00.

The regression analysis for the language status grouping revealed no significant relationship between nonverbal WM and *A'* scores for the NL group on early errors, *F*(1,52) = 3.69, *p′* = .12, *R* ^*2*^ = 0.07. However, there was a significant association between WM and *A’* scores for the NL group for late errors, *F*(1,52) = 12.65, *p’* = .00, *R* ^*2*^ = 0.20, with the N-back task performance predicting morphosyntactic processing. The opposite profile was observed for the LI group, such that there was a significant relationship between WM and *A’* scores on the grammatical judgment task for early errors, *F*(1,28) = 7.62, *p’* = .03, *R* ^*2*^ = 0.21, but no significant association for late errors, *F*(1,28) = 0.72, *p′* = .41, *R* ^*2*^ = 0.03.

With respect to RT scores for the language status groups, there was not a significant relationship between WM and performance for the NL group on early error items, *F*(1,52) = 4.98, *p’* = .10, *R* ^*2*^ = 0.09, or on late errors, *F*(1,52) =5.42, *p’* = .10, *R* ^*2*^ = 0.09. Likewise, for the LI group, there was no significant relationship between WM and RT on early errors, *F*(1,27) = 0.91, *p’* = .70, *R* ^*2*^ = 0.03, or late errors, *F*(1,28) = 0.01, *p’* = .92, *R* ^*2*^ = 0.00.

## Discussion

In this study, we examined the relationship between nonverbal WM and morphosyntactic processing as indexed by a grammatical judgment task in school-age children defined in terms of diagnostic category or language status. Groups did not differ significantly in their performance on an N-back task used to assess nonverbal WM. As discussed below, there were significant group differences in sensitivity to errors, but not in reaction time or response bias on the grammatical judgment measure. Children’s sensitivity to errors was contingent on the position of the errors in the sentence such that detection of late errors surpassed that of early errors, except for the NL group (who performed equally well on early and late errors). Additionally, children in all groups responded more quickly to sentences with late errors than early errors. Our findings revealed that nonverbal WM significantly predicted morphosyntactic processing (sensitivity to errors and/or reaction time), but that the specific profile of associations differed for groups who varied with respect to diagnostic category or language status.

### Grammatical judgment task performance

When morphosyntactic processing was compared on a grammatical judgment task across groups of children from different diagnostic categories (TD, ASD, SLI) who were comparable on age, nonverbal IQ, and SES, only the TD group significantly outperformed the SLI group. This finding is consistent with research indicating that morphosyntactic deficits are a hallmark of SLI, particularly for English speaking children [1]. The ability of the ASD group to detect grammatical errors was intermediate to that of the TD and SLI groups, but was not significantly different from either of those groups. Prior research focused on children with ASD has produced mixed results when considering different morphological structures and comparison groups [3, 34, 36]. Although some investigators have emphasized grammatical difficulties observed in ASD [32], there is considerable evidence of a subgroup of children with ASD who do not have clinically meaningful structural language impairments [2, 101, 102]. Therefore, it is not entirely surprising that a sample of ASD children with normal range cognition who had varying profiles of language skills were not significantly less adept at morphosyntactic processing than typical peers but also not significantly better than children with SLI. There was no significant difference across the diagnostic groups with regard to response bias or the speed with which children correctly responded to sentences on the grammatical judgment task. At first glance this might seem to contradict evidence of “generalized slowing” for children with SLI [103, 104]; however, those findings typically relate to tasks for which accuracy is very high and group differences are observed in reaction times rather than accuracy.

In the current study, results from the diagnostic group analysis revealed that children, regardless of diagnosis, were significantly better at detecting errors that occurred late in the sentence than early in the sentence. Similarly, reaction times (on correct responses) for late errors were faster than for early errors. The findings that both the ASD and SLI groups, as well as the TD group, were better at detecting late occurring errors than early errors are consistent with those of Blackwell and Bates [78] and Wulfeck et al. [28], lending support to the wrap-up account rather than the WM account. For the ASD group, these results contrast with findings by Eigsti and Bennetto [36] who found that children and adolescents with ASD were significantly less sensitive than matched controls to morphosyntactic errors that occurred late in long (but not shorter) sentences, which they attributed to possible deficits in WM. In addition to increased WM load, children with ASD might have been hypothesized to be less sensitive to late errors based on the need to integrate more material and the increased demands for employing contextual information. However, the opposite pattern was found in the current study, providing no evidence based on this verbal task that the ASD group had difficulties with central coherence. This result aligns with other studies with individuals with ASD that have failed to support the weak central coherence theory [101, 105, 106].

When interpreting the findings in terms of language status instead of diagnostic category, the NL group performed better on the grammatical judgment task than the LI group, as would be expected. The LI group was less sensitive to errors when they were early in the sentence, compared to when errors were late in the sentence. This finding in the LI group lends support to the wrap-up account suggesting that sentence context helps to offset the increased memory load associated with greater amounts of information [78]. These findings are also consistent with other research in children with language impairment without any apparent WM difficulty [28]. Conversely, in children with SLI who also had WM impairment, the reverse relationship has been found; that is, children had greater difficulty detecting early errors, indicating they were less able to benefit from the context of the sentence to reduce WM load [66]. It appears that where the effects of WM load manifest in such grammatical judgment tasks is likely to depend on the child’s underlying language and WM abilities.

In contrast to the LI group, the NL group performed equally well whether the error occurred early or late in the sentence. Recall that both the TD and ASD diagnostic groups were more sensitive to late errors relative to early errors. The NL group was comprised of the TD children plus a subset of the ASD children with normal range structural language (ALN). Therefore, it is not entirely clear how to explain this finding. There is no evidence to suggest that variations in nonverbal WM abilities played a role given that the TD and NL groups displayed very similar levels of performance on the N-back task. It is worth noting that when nonverbal IQ was taken into account in the language status group analysis, the main effect of position was no longer significant, suggesting a connection between overall nonverbal cognition and detection of morphosyntactic errors across varying sentence positions.

### Nonverbal working memory

For both the diagnostic groups and language status groups, there were no significant group differences in performance on the N-back task which was used to assess nonverbal WM. That is, children with SLI or ASD in this study demonstrated nonverbal WM abilities that were equivalent to TD peers. Similarly, children with language impairment (across diagnostic categories) displayed visual WM abilities that were comparable to children without language impairment. Most previous research has examined WM deficits using a diagnostic category grouping, with some studies subdividing ASD groups into those with and without language impairment [e.g., 14]. The present results are consistent with studies that have failed to find deficits in visuospatial WM for children with SLI [67, 107, 108]. However, they run counter to other findings of deficits in nonverbal WM in SLI [40, 45]. Current results concur with investigations that report normal nonverbal WM in children with ASD [14, 52], but are inconsistent with findings of deficits in nonverbal WM [47–49]. Differences in various child characteristics (age, IQ, severity of ASD, language impairment, etc.), task demands, and nature of the nonverbal stimuli across these studies likely contributed to the inconsistent findings. Prior research has pointed to a strong connection between CE components of WM and fluid intelligence [57]. Given that the diagnostic groups in the present study had been selected to be comparable on nonverbal intelligence (based on the Perceptual Reasoning Index from the WISC-IV), this likely constrained the variability in WM within the SLI and ASD groups such that no group differences were found on the N-back task.

### Relation between nonverbal WM and morphosyntactic processing

Results from the current study demonstrated that individual differences in nonverbal WM predicted how accurately and/or quickly children detected morphosyntactic errors. For the groups without language impairments (TD and NL), nonverbal WM predicted detection of late errors, whereas for the groups with language impairments (SLI and LI), WM did not predict sensitivity to late errors. Instead, nonverbal WM predicted sensitivity to early errors for the LI group. The finding that WM did not predict sensitivity to morphosyntactic errors for the SLI group conflicts with prior research [66]. It is possible that these contrasting findings stem from Noonan and colleagues’ use of a composite WM measure involving a verbal task in addition to visuospatial WM tasks compared to the use of only a nonverbal WM task in the current study. However, it seems more likely that the nonsignificant finding for the SLI group in the current study was related to low performance on the grammatical judgment task, lack of variance, and the relatively small sample size. For the ASD group WM predicted detection of early and late errors. This was the most heterogeneous group which included children with and without language impairments, with the widest range of performance in grammatical judgment. Nonverbal WM predicted RT for late errors only for the TD group. For the groups including children with language impairments (SLI, LI, and ASD) the link between WM and morphosyntactic processing was reflected in accuracy rather than speed of processing. This suggests that the language system of children with typical, but not impaired, language is characterized by an interdependence between speed of morphosyntactic processing and nonverbal WM. It is possible that the absence of the link between nonverbal WM and RTs in the diagnostic groups and the LI group was due to the fact that these groups found the grammatical judgment more challenging, and thus prioritized accuracy over efficiency in their responses. The results of the sensitivity data appear to support this interpretation.

According to the wrap-up account [78], late errors should be detected with greater ease than early errors because the availability of contextual information offsets WM demands associated with parsing a sentence as it unfolds. It is notable, however, that such a relationship between WM and error-processing in different sentence positions was only hypothesized by prior studies, but never empirically tested. Our findings for the children with typical development indicate that while late errors were indeed detected with greater sensitivity than early errors in a sentence, the ability to detect late errors relied on nonverbal WM resources more so than the ability to detect early errors. That is, integration of contextual information during morphosyntactic processing associated with late-error processing appears to require a greater reliance on WM. In this regard, the findings for the children with typical language skills also partially align with the WM account [66], which posits that detection of late errors should rely on WM more than detection of early errors. We had hypothesized that the relationship between nonverbal WM and morphosyntactic processing would be stronger for children with language impairment than for children with normal language. However, this prediction was not borne out. Nonverbal WM was implicated in grammatical processing across groups, but the specific pattern of association between WM and early versus late error detection varied according to language ability.

In summary, findings revealed that the SLI and LI groups were less accurate and slower at detecting grammatical errors than their peers but yet displayed similar patterns of error detection to typically developing children. Despite limitations in linguistic knowledge in the children with language impairment, they nevertheless were able to take advantage of wrap-up effects of accumulating sentence context as indexed by better performance on late than early errors. In this study the SLI and ASD children, unlike some children with these diagnostic labels, did not demonstrate complex nonverbal WM deficits. Similarly, the LI group did not have poorer nonverbal WM than the NL group. While there is no indication that nonverbal WM is a causal factor in the language deficits in these children, findings suggest that it does play a role in their *processing* of linguistic stimuli. Nonverbal WM predicted early errors for the LI group but late errors for the NL group. We speculate that children in the LI group could use context to aid in detection of late errors but relied more heavily on generalized controlled attention within WM to detect early errors (for which less contextual support was available) given their more limited grammatical abilities. On the other hand, nonverbal WM predicted detection of morphosyntactic errors for the NL and TD children who had strong linguistic skills only when errors occurred later in the sentence and there was more linguistic material to keep in mind, perhaps implicating updating components of nonverbal WM rather than attention allocation. Further research is needed to explore these speculations and to establish how children’s level of grammatical proficiency relates to the interplay between accrual of context and recruitment of executive attention and WM during morphosyntactic processing.

The current study provides new insights into the relation between nonverbal WM and morphosyntactic processing for children with varying clinical diagnoses and language abilities. There are, however, certain limitations of this investigation that must be acknowledged. These findings only apply to relatively more able children on the autism spectrum who are verbal and do not have co-morbid intellectual disabilities. While some of the children within the ASD group had language impairment, all of the children had normal-range nonverbal cognitive abilities and behavioral control that enabled them to complete the various language assessments and computer-based experimental tasks. Another limitation of this study is that the grammatical judgment task focused only on omissions of auxiliary forms and regular past tense markers. Future work could expand the variety of grammatical structures as well as the types of error patterns to gain a more comprehensive understanding of morphosyntactic processing in children with SLI and children with ASD with and without structural language deficits. Finally, the diagnostic groups were not matched in terms of sex, so findings could have been influenced by this factor. However, the high proportion of males in the ASD group (85%) reflects disproportionate sex ratios in the population, whereas the TD and SLI groups were balanced (58 and 57%, respectively). The NL and LI groups both consisted of 63% males.

## Conclusion

It is well known that children with language impairment are not as adept as those with typical language in detecting morphosyntactic errors. Although our findings confirm this, our main objective was to examine the error position effects on a grammatical judgment task in accordance with distinct predictions about underlying mechanisms involved in different patterns of error detection. Our study is the first cross-group (SLI and ASD) comparison of the WM and wrap-up accounts of morphosyntactic processing. On the whole, results from the grammatical judgment task indicated that children in all groups were more accurate and faster at detecting errors occurring late, rather than early, in the sentence—supporting the wrap-up account. Thus, there was no evidence of different patterns of performance for the SLI and ASD groups with respect to early/late error detection (even though children with SLI were significantly less proficient than children with typical language). Despite this broad support for the wrap-up account, WM abilities (in this sample of children without nonverbal WM deficits) were found to differentially predict morphosyntactic processing for children with and without language impairment using a novel approach to examining language status that was “diagnosis neutral”. Taken together, the results indicate that cognitive processes supporting both integration of accumulating context and nonverbal WM are involved in grammatical judgment. Therefore, a more nuanced framework of morphosyntactic processing seems to be warranted.

## Additional file

Additional file 1: Sample Grammatical Judgment Task Stimuli. (DOC 26 kb)

## Abbreviations

ASD: Autism spectrum disorders
LI: Language impairment (SLI plus ASD with structural language deficits)
NL: Normal language (TD plus ASD without structural language deficits)
SLI: Specific language impairment
TD: Typically developing controls with no neurodevelopmental disorders
WM: Working memory

## References

[1] Leonard L (2014). Children with specific language impairment.

[2] Kjelgaard M, Tager-Flusberg H (2001). An investigation of language impairment in autism: implications for genetic subgroups. Lang Cogn Process.

[3] Roberts J, Rice M, Tager-Flusberg H (2004). Tense marking in children with autism. Appl Psycholinguist.

[4] Montgomery J, Magimairaj B, Finney M (2010). Working memory and specific language impairment: an update on the relation and perspectives on assessment and treatment. Am J Speech-Language Pathol.

[5] Archibald L, Gathercole S (2006). Short-term and working memory in specific language impairment. Int J Commun Disord.

[6] Archibald L, Gathercole S (2007). The complexities of complex memory span: storage and processing deficits in specific language impairment. J Mem Lang.

[7] Gabig C (2008). Verbal working memory and story retelling in school-age children with autism. Lang Speech Hear Serv Sch.

[8] Steele S, Minshew N, Luna B, Sweeney J (2007). Spatial working memory deficits in autism. J Autism Dev Disord.

[9] Atkins P, Baddeley A (1998). Working memory and distributed vocabulary learning. Appl Psycholinguist.

[10] Daneman M, Carpenter P (1980). Individual differences in working memory and reading. J Verbal Learning Verbal Behav.

[11] King J, Just M (1991). Individual differences in syntactic processing: the role of working memory. J Mem Lang.

[12] McDonald J (2008). Grammaticality judgments in children: the role of age, working memory and phonological ability. J Child Lang.

[13] Finney M, Montgomery J, Gillam R, Evans J (2014). Role of working memory storage and attention focus switching in children’s comprehension of spoken object relative sentences. Child Dev Res.

[14] Hill A, van Santen J, Gorman K, Langhorst B, Fombonne E (2015). Memory in language-impaired children with and without autism. J Neurodev Disord.

[15] Montgomery J (2000). Relation of working memory to off-line and real-time sentence processing in children with specific language impairment. Appl Psycholinguist.

[16] Montgomery J (2000). Verbal working memory and sentence comprehension in children with specific language impairment. J SPeech, Lang Hear Res.

[17] Montgomery J, Gillam R, Evans J (2016). Syntactic versus memory accounts of the sentence comprehension deficits of specific language impairment: looking back, looking ahead. J Speech Lang Hear Res.

[18] MacDonald M, Christiansen M (2002). Reassessing working memory: comment on just and carpenter (1992) and waters and Caplan (1996). Psychol Rev.

[19] Vergauwe E, Barrouillet P, Camos V (2010). Do mental processes share a domain-general resource?. Psychol Sci.

[20] Rice M, Levy Y, Schaeffer J (2003). A unified model of specific and general language delay: grammatical tense as a clinical marker of unexpected variation. Language across populations: towards a definition of specific language impairment.

[21] Rice M, Wexler K (1996). Toward tense as a clinical marker of specific language impairment in English-speaking children. J Speech Lang Hear Res.

[22] Tager-Flusberg H, Cooper J (1999). Present and future possibilities for defining a phenotype for specific language impairment. J Speech Lang Hear Res.

[23] Conti-Ramsden G, Windfuhr K (2002). Productivity with word order and morphology: a comparative look at children with SLI and children with normal language abilities. Int J Lang Commun Disord.

[24] Leonard L, Deevy P, Miller C, Rauf L, Charest M, Robert K (2003). Surface forms and grammatical functions: past tense and passive participle use by children with specific language impairment. J Speech Lang Hear Res.

[25] Leonard L, Miller C, Owen A (2000). The comprehension of verb agreement morphology by English-speaking children with specific language impairment. Clin Linguist Phon.

[26] Marchman V, Wulfeck B, Ellis Weismer S. Morphological productivity in children with normal language and SLI: a study of the English past tense. J Speech Lang Hear Res. 1999;42:206–19. 10.1044/jslhr.4201.206 10025555

[27] Redmond S (2005). Differentiating SLI, from ADHD using children’s sentence recall and production of past tense morphology. Clin Linguist Phon.

[28] Wulfeck B, Bates E, Krupa-Kwiatkowski M, Saltzman D (2004). Grammaticality sensitivity in children with early focal brain injury and children with specific language impairment. Brain Lang.

[29] Bedore L, Leonard L (1998). Specific language impairment and grammatical morphology: a discriminant function analysis. J Speech Lang Hear Res.

[30] Leonard L, Eyer J, Bedore L, Grela B (1997). Three accounts of the grammatical morpheme difficulties of English-speaking children with specific language impairment. J Speech Lang Hear Res.

[31] Rice M, Wexler K, Cleave P (1995). Specific language impairment as a period of extended optional infinitive. J Speech Hear Res.

[32] Eigsti I, de Marchena A, Schuh J, Kelley E (2011). Language acquisition in autism spectrum disorders: a developmental review. Res Autism Spectr Disord.

[33] Eigsti I, Bennetto L, Dadlani M (2007). Beyond pragmatics: morphosyntactic development in autism. J Autism Dev Disord.

[34] Botting N, Conti-Ramsden G (2003). Autism, primary pragmatic difficulties, and specific language impairment: can we distinguish them using psycholinguistic markers?. Dev Med Child Neurol.

[35] American Psychiatric Association (2013). Diagnostic and statistical manual of mental disorders.

[36] Eigsti I, Bennetto L (2009). Grammaticality judgments in autism: deviance or delay. J Child Lang.

[37] Baddeley A (2003). Working memory and language: an overview. J Commun Disord.

[38] Ellis Weismer S, Evans J, Hesketh L (1999). An examination of verbal working memory capacity in children with specific language impairment. J Speech Lang Hear Res.

[39] Ellis Weismer S, Tomblin J, Zhang X, Buckwhalter P, Chynoweth J, Jones M (2000). Nonword repetition performance in school-age children with and without language impairment. J Speech Lang Hear Res.

[40] Vugs B, Hendriks M, Cuperus J, Verhoeven L (2014). Working memory performance and executive function behaviors in young children with SLI. Res Dev Disabil.

[41] Bavin E, Wilson P, Maruff P, Sleeman F (2005). Spatiovisual memory of children with specific language impairment: evidence for generalized processing problems. Int J Lang Commun Disord.

[42] Henry L, Messer D, Nash G (2012). Executive functioning in children with specific language impairment. J Child Psychol Psychiatry..

[43] Marton K (2008). Visuospatial processing and executive functions in children with specific language impairment. Int J Lang Commun Disord.

[44] Im-Bolter N, Johnson J, Pascual-Leone J (2006). Processing limitations in children with specific language impairment: the role of executive function. Child Dev.

[45] Vugs B, Cuperus J, Hendriks M, Verhoeven L (2013). Visuospatial working memory in specific language impairment: a meta-analysis. Res Dev Disabil.

[46] Vugs B, Knoors H, Cerus J, Hendricks M, Verhoeven L (2016). Interactions between working memory and language in young children with specific language impairment (SLI). Child Neuropsychol.

[47] Joseph R, McGrath L, Tager-Flusberg H (2005). Executive dysfunction and its relation to language ability in verbal school-age children with autism. Dev Neuropsychol.

[48] Williams D, Goldstein G, Carpenter P, Minshew N (2005). Verbal and spatial working memory in autism. J Autism Dev Disord.

[49] Williams D, Goldstein G, Minshew N (2005). Impaired memory for faces and social scenes in autism: clinical implications of memory dysfunction. Arch Clin Neuropsychol.

[50] Schuh J, Eigsti I (2012). Working memory, language skills, and autism symptomatology. Behav Sci (Basel).

[51] Williams D, Goldstein G, Minshew N (2006). The profile of memory function in children with autism. Neuropsychology.

[52] Happé F, Booth R, Charlton R, Hughes C (2006). Executive function deficits in autism spectrum disorders and attention-deficit/hyperactivity disorder: examining profiles across domains and ages. Brain Cogn.

[53] Baddeley A, Baddeley A (1986). The articulatory loop. Working memory.

[54] Barrouillet P, Camos V (2010). Working memory and executive control: a time-based resource-sharing account. Psychol Belg.

[55] Cowan N (1995). Attention and memory: an integrated framework.

[56] Cowan N, Elliott E, Saults S, Morey C, Mattox S, Hismjatullina A (2005). On the capacity of attention: its estimation and its role in working memory and cognitive aptitudes. Cogn Psychol.

[57] Engle R, Tuholski S, Laughlin J, Conway A (1999). Working memory, short-term memory, and general fluid intelligence: a latent-variable approach. J Exp Psychol Gen.

[58] Just M, Carpenter P (1992). A capacity theory of comprehension: individual differences in working memory. Psychol Rev.

[59] Miyake A, Shah P (1999). Model of working memory: mechanisms of active maintenance and executive control.

[60] Baddeley A (1996). The fractionation of working memory. Proc Natl Acad Sci U S A.

[61] Courage M, Cowan N (2009). Development of WM in infancy and childhood.

[62] Engle R (2002). Working memory capacity as executive attention. Curr Dir Psychol Sci.

[63] Barrouillet P, Bernardin S, Camos V (2004). Time constraints and resource sharing in adults’ working memory spans. J Exp Psychol Gen.

[64] Kane M, Hambrick D, Tuholski S, Wilhelm O, Payne T, Engle R (2004). The generality of working memory capacity: a latent-variable approach to verbal and visuospatial memory span and reasoning. J Exp Psychol Gen.

[65] Magimairaj B, Montgomery J (2012). Children’s verbal working memory: relative importance of storage, general processing speed, and domain-general controlled attention. Acta Psychol (Amst).

[66] Noonan N, Redmond S, Archibald L (2014). Contributions of children’s linguistic and working memory proficiencies to their judgments of grammaticality. J Speech Lang Hear Res.

[67] Baird G, Dworzynski K, Slonims V, Simonoff E (2010). Memory impairment in children with language impairment. Dev Med Child Neurol.

[68] Montgomery J, Evans J (2009). Complex sentence comprehension and working memory in children with specific language impairment. J Speech Lang Hear Res.

[69] Marton K, Schwartz R (2003). Working memory capacity and language processes in children with specific language impairment. J Speech Lang Hear Res.

[70] Marton K, Schwartz R, Farkas L, Katsnelson V (2006). Effect of sentence length and complexity on working memory performance in Hungarian children with specific language impairment (SLI): a cross-linguistic comparison. Int J Commun Disord.

[71] Leonard L, Ellis Weismer S, Miller C, Francis D, Tomblin J, Kail R (2007). Speed of processing, working memory, and language impairment in children. J Speech Lang Hear Res.

[72] Karasinski C, Ellis Weismer S. Comprehension of inferences in discourse processing by adolescents with and without language impairment. J Speech Lang Hear Res. 2010;53:1268–79. 10.1044/1092-4388(2009/09-0006) PMC3064077 20631230

[73] Landa R, Goldberg M (2005). Language, social, and executive functions in high functioning autism: a continuum of performance. J Autism Dev Disord.

[74] Gangopadhyay I, Davidson M, Ellis Weismer S, Kaushanskaya M (2016). The role of nonverbal working memory in morphosyntactic processing by school-aged monolingual and bilingual children. J Exp Child Psychol.

[75] Wulfeck B (1993). A reaction time study of grammaticality judgements in children. J Speech Lang Hear Res.

[76] Hayiou-Thomas M, Bishop D, Simulating PK, SLI (2004). General cognitive specific linguistic profile. J Speech Lang Hear Res.

[77] Salamé P, Baddeley A (1987). Noise, unattended speech and short-term memory. Ergonomics.

[78] Blackwell A, Bates E (1995). Inducing agrammatic profiles in normals: evidence for the selective vulnerability of morphology under cognitive resource limitation. J Cogn Neurosci.

[79] Ellis Weismer, S. Developmental language disorders: challenges and implications of cross-group comparisons. Folia Phoniatr Logop. 2013;65:68–77. 10.1159/000353896 PMC4004334 23942044

[80] Rice M (2016). Specific language impairment, nonverbal IQ, attention-deficit/hyperactivity disorder, autism spectrum disorder, cochlear implants, bilingualism, and dialectal variants: defining the boundaries, clarifying clinical conditions, and sorting out causes. J Speech Lang Hear Res.

[81] Tager-Flusberg H (2016). Risk factors associated with language in autism spectrum disorder: clues to underlying mechanisms. J Speech Lang Hear Res.

[82] Williams D, Payne H, Marshall C (2013). Non-word repetition impairment in autism and specific language impairment: evidence for distinct underlying cognitive causes. J Autism Dev Disord.

[83] Frith U. Autism: explaining the enigma. Oxford: Blackwell Publishers: 1989.

[84] Happé F, Frith U (2006). The weak coherence account: detail-focused cognitive style in autism spectrum disorders. J Autism Dev Disord.

[85] Haebig E, Kaushanskaya M, Ellis Weismer S. Lexical processing in school-age children with autism spectrum disorder and children with specific language impairment: the role of semantics. J Autism Dev Disord. 2015;45:4109–23. 10.1007/s10803-015-2534-2 PMC4761424 26210517

[86] Wechsler D, Kaplan E, Fein D, Kramer J, Morris R, Delis D (2003). Wechsler intelligence scale for children.

[87] Schopler E, Van Bourgondien M, Wellman G, Love S (2010). Childhood autism rating scale, second edition. Second.

[88] Rutter M, Bailey A, Lord C (2003). The social communication questionnaire.

[89] Semel E, Wiig E, Secord W (2003). Clinical evaluation of language fundamentals.

[90] Schneider W, Eschman A, Zuccolotto A (2002). E-Prime user’s guide.

[91] Smith E, Jonides J (1999). Storage and executive processes in the frontal lobes. Science (80-)..

[92] Szmalec A, Verbruggen F, Vandierendonck A, Kemps E (2011). Control of interference during working memory updating. J Exp Psychol.

[93] Attneave F, Arnoult M (1956). The quantitative study of shape and pattern perception. Psychol Bull.

[94] Vanderplas J, Garvin E (1959). The association value of random shapes. J Exp Psychol.

[95] Rice M (1996). Toward a genetics of language.

[96] Rice M, Wexler K, Redmond S (1999). Grammaticality judgments of an extended optional infinitive grammar evidence from English speaking children with specific language impairment. J Speech Lang Hear Res.

[97] Stanislaw H, Todorov N (1999). Calculation of signal detection theory measures. Behav Res Methods.

[98] Dennis M, Francis DJ, Cirino P, Schachar R, Barnes M, Fletcher J. Why IQ is not a covariate in cognitive studies of neurodevelo9pmentla disorders. J. Int. Neurpsychol. Soc. 2009;15:331–43. 10.1017/S1355617709090481 PMC3075072 19402919

[99] Gaetano J (2013). Holm-Bonferroni sequential correction: an EXCEL calculator [Microsoft Excel workbook].

[100] Kaushanskaya M, Park J, Gangopadhyay I, Davidson M, Ellis Weismer S. The relationship between executive functions and language abilities in children: a latent variables approach. J Speech Lang Hear Res. 2017;60:912–23. 10.1044/2016_JSLHR-L-15-0310 PMC5548084 28306755

[101] Norbury C (2005). Barking up the wrong tree? Lexical ambiguity resolution in children with language impairments and autistic spectrum disorders. J Exp Child Psychol.

[102] Tager-Flusberg H (2006). Defining language phenotypes in autism. Clin Neurosci Res.

[103] Kail R (1994). A method for studying the generalized slowing hypothesis in children with specific language impairment. J Speech Lang Hear Res.

[104] Miller C, Kail R, Leonard L, Tomblin J (2001). Speed of processing in children with specific language impairment. J Speech Lang Hear Res.

[105] Brock J, Norbury C, Einav S, Nation K (2008). Do individuals with autism process words in context? Evidence from language-mediated eye-movements. Cognition.

[106] Hahn N, Snedeker J, Rabagliati H (2015). Rapid linguistic ambiguity resolution in young children with autism spectrum disorder: eye tracking evidence for the limits of weak central coherence. Autism Res.

[107] Alloway T, Archibald L (2008). Working memory and learning in children and specific language impairment. J Learn Disabil.

[108] Williams D, Stott C, Goodyer I, Sahakian B (2000). Specific language impairment with or without hyperactivity: Neuropsychological evidence for frontostriatal dysfunction. Dev Med Child Neurol.

