# Supplementary material for: The role of nonverbal working memory in morphosyntactic processing by children with specific language impairment and autism spectrum disorders
Source: J Neurodev Disord. 2017 Jul 4;9:28. doi: 10.1186/s11689-017-9209-6 (PMC5496437; doi:10.1186/s11689-017-9209-6)
Supplement: Additional file 1: — Sample Grammatical Judgment Task Stimuli. (DOC 26 kb) [file 11689_2017_9209_MOESM1_ESM.doc]

**Appendix: Sample Grammatical Judgment Task Stimuli**

Auxiliary

Early Control

*They* ***were*** *sharing several comic books while waiting for the next bus.*

Early Omission Error

*The children ____ asking if they could go outside at recess.**

Late Control

*The Nelsons’ two black labs* ***were*** *barking last night.*

Late Omission Error

*Even though they had both practiced the song, they ____ worrying about tonight.**

Regular Past Tense

Early Control

*While Chloe* ***looked*** *at her new book, she sat on the couch.*

Early Omission Error

*While Jacob* ***wait__*** *for his friends, he played his Nintendo DS.**

Late Control

*While he was listening to music, he* ***tapped*** *his foot.*

Late Omission Error

*Last evening in a beautiful park a girl* ***pick****__ some flowers.**
